# Co-occurrence of Local Anisotropic Gradient Orientations (CoLlAGe): A new radiomics descriptor

**DOI:** 10.1038/srep37241

**Published:** 2016-11-22

**Authors:** Prateek Prasanna, Pallavi Tiwari, Anant Madabhushi

**Affiliations:** 1 Department of Biomedical Engineering, Case Western Reserve University, Cleveland, OH 44120, USA

## Abstract

In this paper, we introduce a new radiomic descriptor, Co-occurrence of Local Anisotropic Gradient Orientations (CoLlAGe) for capturing subtle differences between benign and pathologic phenotypes which may be visually indistinguishable on routine anatomic imaging. CoLlAGe seeks to capture and exploit local anisotropic differences in voxel-level gradient orientations to distinguish similar appearing phenotypes. CoLlAGe involves assigning every image voxel an entropy value associated with the co-occurrence matrix of gradient orientations computed around every voxel. The hypothesis behind CoLlAGe is that benign and pathologic phenotypes even though they may appear similar on anatomic imaging, will differ in their local entropy patterns, in turn reflecting subtle local differences in tissue microarchitecture. We demonstrate CoLlAGe’s utility in three clinically challenging classification problems: distinguishing (1) radiation necrosis, a benign yet confounding effect of radiation treatment, from recurrent tumors on T1-w MRI in 42 brain tumor patients, (2) different molecular sub-types of breast cancer on DCE-MRI in 65 studies and (3) non-small cell lung cancer (adenocarcinomas) from benign fungal infection (granulomas) on 120 non-contrast CT studies. For each of these classification problems, CoLlAGE in conjunction with a random forest classifier outperformed state of the art radiomic descriptors (Haralick, Gabor, Histogram of Gradient Orientations).

There are several instances where benign and malignant pathologies might appear very similar on radiographic imaging. One such example is radiation necrosis (RN) (a relatively benign effect of radiation treatment) and recurrent brain tumors (rBT), which are visually almost indistinguishable on conventional MRI^1^; even though both RN and rBT have distinct cellular and architectural arrangements when examined on a pathology slide under a microscope. Another example is triple negative (TN) breast cancer (highly aggressive) and fibroadenomas (FA) (benign tumor) with similar morphologic appearances on MRI^2^. Similarly, fungal infections known as granulomas look strikingly similar to non-small cell lung cancers (adenocarcinomas) on routine non-contrast CT imaging. There is hence a need for identifying non-invasive markers that can reliably distinguish such similar appearing pathologies on routine imaging for early diagnosis as well as treatment evaluation. Identification of these imaging biomarkers could potentially obviate the need for unnecessary surgical interventions, as well as exposure to unnecessary radiation, for disease confirmation. “Radiomics”^3^,^4^,^5^, an emerging field in medical image analysis, refers to the quantitative extraction of shape, histogram, and/or texture-based features from radiographic images to distinguish disease phenotypes that are not visually appreciable on imaging. Two popular radiomic features are Haralick^6^ and Gabor steerable filters^7^. Haralick features capture gray-level co-occurrence patterns^8^,^9^, where a matrix of co-occurring gray-level pairs in the image is constructed, from which second-order statistical texture features can be calculated. Haralick texture analysis is relatively popular in medical image analysis as it allows for capturing variations in gray-level image characteristics via second order intensity statistics (e.g. angular second moment, contrast, and difference entropy). However, Haralick features may fail to capture variations in subtly different sub-structures that may be morphologically different but may have identical co-occurring gray-level intensities. Figure 1(b) and (f) show one such example of two similar appearing texture patterns, where the corresponding Haralick energy feature (shown in Fig. 1(c) and (g)) for both the patterns was found to be identical.

Gabor filters^7^ are modeled to mimic the way human visual system deciphers object appearances^10^. A Gabor filter can be defined as the modulation of a complex sinusoidal by a Gaussian function and is controlled by scale (*t*) and orientation (*λ*) parameters. Gabor features can be extracted as a response to convolution of an image with distinct Gabor filters obtained by varying each of the associated parameters (*t, λ*) across the filter bank. However, Gabor filters capture a global response for an image at a specific value of (*t, λ*), and may not capture local variations in orientations on a per-pixel basis within local neighborhoods, which may be an important attribute when computing differences across similar-appearing micro-textures. Figure 1(d) and (h) show a representative example of similar feature responses obtained for a Gabor filter at *t* = 2, and *λ* = *π*/4 for the two texture patterns shown in Fig. 1(b) and (f) respectively.

Other popular radiomic features include histogram of gradient orientations (HOG)^11^. HOG yields a global patch-based signature by computing histogram distribution of orientations obtained from computing differences in image intensities in *X* and *Y* directions on a per pixel basis. A variant of HOG, called co-occurrence of histogram of gradient orientations (Co-HOG) was recently presented by Watanabe *et al*.^12^ and Pang *et al*.^13^ for pedestrian detection. Co-HOG is a multiple-gradient-orientation-based feature descriptor. Its computation yields a high dimensional feature vector that combines neighbor gradient orientations to quantify shape-based appearances in regions of interest. However, the Co-HOG approach in refs 12, 13, 14 (a) does not capture localized intensity-dependent variations across neighboring orientations, and (b) is susceptible to “curse of dimensionality” (due to a high dimensional feature space).

Recently, Lee *et al*.^15^ developed a novel quantitative feature called cell orientation entropy (CoRE) to capture differences in orientation of nuclei with respect to the neighboring nuclei across different pathologies, and demonstrated differences in nuclear orientations across aggressive and benign conditions in the context of prostate cancer. If such pathologic differences are indeed reflected at the radiologic level (even though these differences may not be visually discernible), it begs the question whether a radiomic descriptor could be developed to capture the histologic anisotropy across different pathologies at the radiologic scale.

In this work, we present a new radiomic descriptor, Co-occurrence of Local Anisotropic Gradient Orientations (CoLlAGe), to capture anisotropic tensor gradient differences across similar appearing pathologies in an image. The rationale behind CoLlAGe, is that even though overall the global textural patterns or even the filter responses at a majority of pixel locations might be similar between two differing pathological conditions (e.g. RN versus rBT, FA versus TN breast cancer or adenocarcinomas versus granulomas), the organization and co-occurrences of local tensor gradients may differ across classes and will be relatively consistent within a class. CoLlAGe seeks to capture these local anisotropic differences in micro-structures by measuring entropy (a mathematical construct to measure disorder) of co-occurrences of pixel/voxel-level gradient orientations computed within a local neighborhood. The rationale being that the distribution of entropy of localized gradient field within a lesion will be high for aggressive disease conditions, potentially manifesting their inherent disorder and high heterogeneity appreciable at a cellular scale, as compared to benign pathologies which have a more coherent micro-architecture. An example of CoLlAGe is shown in Fig. 1(e) and (i) to distinguish two synthetic checkerboard images with similar-appearing patterns. CoLlAGe was found to capture localized variations in gradients on a per-pixel basis (reflected by high CoLlAGe values) across the two patterns, differences that were not appreciable on Haralick and Gabor feature representations.

The rest of the paper is organized as follows. The algorithm of CoLlAGe is detailed in the Methodology section, followed by the experimental setup to demonstrate the utility of CoLlAGe in the context of three problems involving brain tumors, breast cancer, and lung cancer. Subsequently we present and discuss the results followed by the concluding remarks.

## Methodology of CoLlAGe

**Figure 2:**
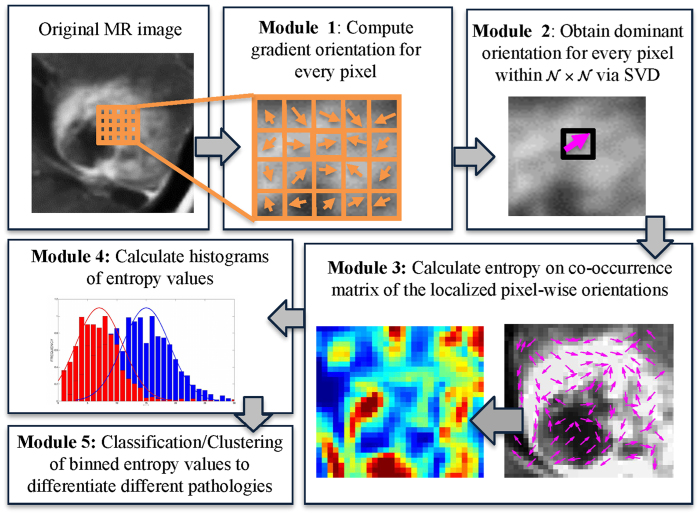
Overview of 2-dimensional CoLlAGe and overall workflow. The first module involves computing gradient orientations on a per-pixel basis within the lesion. In the second module, dominant direction for every pixel is computed within a 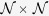 neighborhood via singular value decomposition. A co-occurrence matrix of orientations and subsequently the associated entropy of each pixel is calculated in the third module. A histogram of entropy values is then aggregated for every pixel in the fourth module and used for classification in the fifth module to distinguish similar appearing pathological conditions.

**Figure 3:**
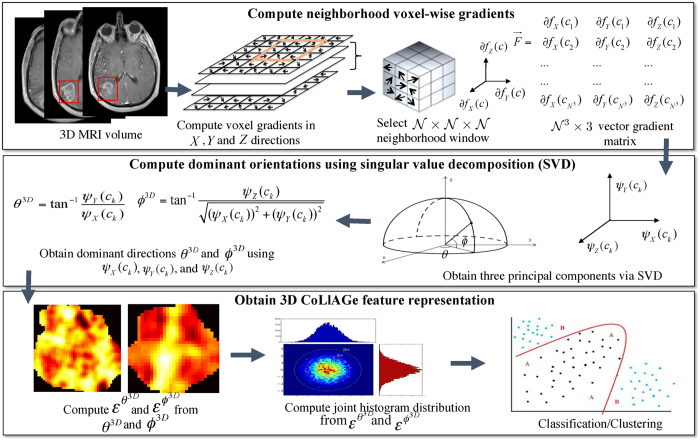
Workflow for extracting 3D CoLlAGe features. Note that unlike 2D features, two dominant directions (*θ*^3*D*^, *ϕ*^3*D*^) are computed for 3D CoLlAGe associated with each *c* ∈ *C*, which provide complimentary information about the degree of disorder of the principal gradient orientations in (*X, Y*) and (*X, Y, Z*) directions.

In the following subsections, we describe the detailed mathematical formulation of our CoLlAGe strategy, both in 2-dimensions (2D) and in 3-dimensions (3D). A preliminary implementation of 2D CoLlAGe was previously presented in ref. 16. Figure 2 shows the workflow of CoLlAGe in 2D, while the 3D implementation is shown in Fig. 3.

### Notation

An MRI image scene 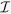 is defined as 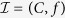 where 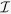 is a spatial grid *C* of locations *c* ∈ *C*, in a 2-dimensional, 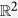, or a 3-dimensional space, 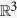. Each such spatial location, *c* ∈ *C* (in 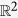 or 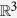) is associated with an intensity value *f(c*). A local neighborhood of 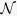 pixels/voxels is defined within a window *W*, while the co-occurrence matrix computed from within *W* is denoted as 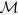. The entropy map is given as 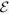, and the final CoLlAGe feature vector is denoted as **F**. For the sake of clarity, the notations used for computation of 2D CoLlAGe are denoted with superscript 2*D*, while the notations for computation of 3D CoLlAGe are denoted with superscript 3*D*. The common notations, operators and acronyms employed in this paper are listed in Table 1.

### Methodology

Computation of 2D CoLlAGe (Algortithm 1) for every *c* ∈ *C* involves the following main steps,

1. **Calculation of gradient magnitudes for every pixel**: For every *c* ∈ *C*, gradients along the *X* and *Y* directions are computed as, 
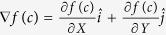
. Here, 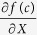 and 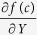 are the gradient magnitudes along the *X* and the *Y* axes respectively, denoted by ∂ *f*_*X*_ (*c*) and ∂ *f*_*Y*_ (*c*).
2. **Computing local dominant orientations via singular value decomposition** (**SVD**): A 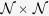 window *W* centered around every *c* ∈ *C* is selected to compute the localized gradient field. We then compute ∂ *f*_*X*_ (*c*_*k*_) and ∂ *f*_*Y*_ (*c*_*k*_), 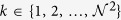. The vector gradient matrix 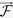 associated with every *c* is given by 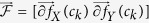, where 
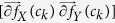
, 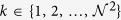 is the matrix of gradient vectors in the *X* and *Y* directions for every *c*_*k*_ given by a 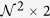 matrix, 
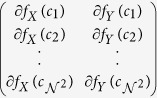
The most significant orientation for each pixel *c*_*k*_ within 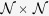 gradient field is obtained by performing singular value decomposition (SVD) of 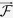. The dominant principal components in *X* and *Y* directions are obtained from SVD as 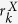 and 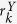 for every 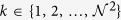. The most significant orientation for every *c*_*k*_ is then calculated as 
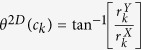
.
3. **Calculation of second**-**order statistics for most significant orientations**: The objects of interest for calculating CoLlAGe features are the co-occurring directions given by discretization of the dominant orientation 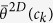 for every pixel *c*, such that 
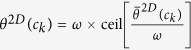
, where *ω* is a discretization factor. An *N* × *N* co-occurrence matrix 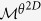 subsequently captures pairs of orientations (*p, q*) between pixels (*c*_*j*_, *c*_*k*_) which co-occur in the neighborhood 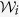, such that, 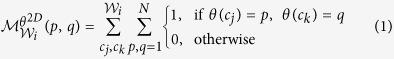 where 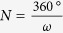 is the number of discrete angular bins. Entropy measure, 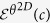 is then computed from every co-occurrence matrix on every *c* as, 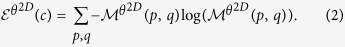
4. A **histogram of** 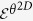 is computed by aggregating 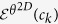, 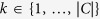, where |·| is the cardinality of set *C*. The entropy histogram is divided into bin size *v*, optimized on the training set via grid search optimization.

A CoLlAGe feature vector, **F**^2*D*^ can be obtained for every *c* ∈ *C* which consists of the binned histogram values in the form of *v* × 1 vectors.


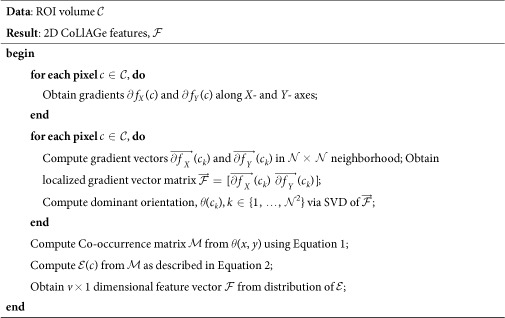


**Algorithm 1**: Computation of 2D CoLlAGe features


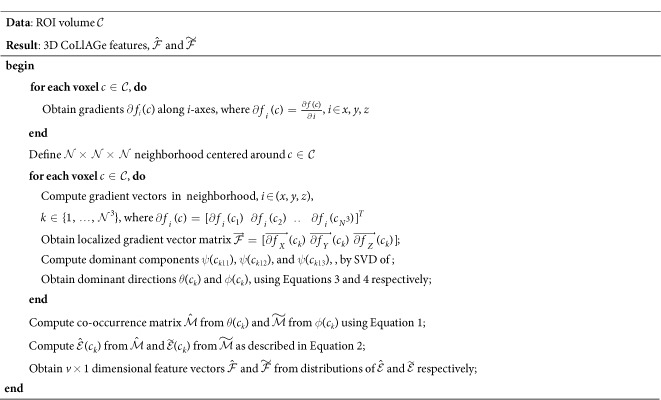


**Algorithm 2**: Computation of 3D CoLlAGe features

### Extension of CoLlAGe to 3-Dimensions

For 3D CoLlAGe (Algortithm 2), the local neighborhood around a voxel is first defined by a 3D window *W* of size 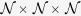 along the *X, Y*, and *Z*-directions and gradient directions for every voxel are calculated from within *W*. We collate the gradient magnitudes along the three axes in *W* into a single gradient matrix 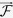 of size 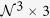 given as: 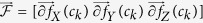, where 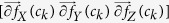, 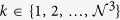 is the matrix of gradient vectors in the *X, Y* and *Z* directions respectively for every *c*_*k*_. SVD of 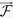 for a voxel *c*_*k*_ yields three dominant principal components *ψ*_*X*_(*c*_*k*_), *ψ*_*Y*_(*c*_*k*_) and *ψ*_*Y*_(*c*_*k*_) in the *X*-, *Y*- and *Z*- directions respectively. Two dominant orientations *θ*^3*D*^(*c*_*k*_) and *ϕ*^3*D*^(*c*_*k*_) can then be obtained from the three principal components to capture variability in orientations across (*X, Y*), and (*X, Y, Z*), given by


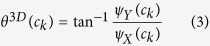


and


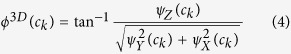


Two co-occurrence matrices 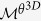 and 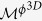 corresponding to *θ*^3*D*^(*c*_*k*_) and *ϕ*^3*D*^(*c*_*k*_) capture the orientation pairs between voxels in the neighborhood and are computed as given in Equation 1. 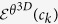 from 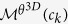 and 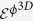 from 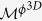 (computed using Equation 2), yield two distinct entropy representations in (*X, Y*) and (*X, Y, Z*) directions within the volume of interest. A joint histogram of CoLlAGe feature vector 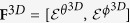 can be obtained for further evaluation in a supervised or an unsupervised classification setting.

## Experimental Design

### Datasets and Preprocessing

In this work, we employed three unique dataset cohorts obtained from different collaborating institutions to evaluate the efficacy of CoLlAGe on three extremely challenging clinical problems: (a) distinguishing radiation necrosis, a relatively benign effect of radiation, from tumor recurrence on T1-w MRI in brain tumors, (b) distinguishing different molecular sub-types of breast cancer on DCE-MRI, and (c) distinguishing adenocarcinomas from granulomas on non-contrast CT images. Details regarding inclusion criteria, pre-processing, and experimental design for each of the three datasets are provided below.

#### Brain tumor dataset

Imaging scans were acquired under an Institutional Review Board (IRB)-approved (IRB # CC00148) and HIPAA-compliant study at University Hospitals, Cleveland (UH). The patient cohorts were identified by performing a retrospective review of neuropathology in all brain tumor patients who underwent a surgery of a recurrent or progressive Gd T1w-enhancing lesion identified during follow-up post-9 months (or later) after the initial after brain radiation therapy. Follow-up MRI scans within 0–21 days prior to second resection or biopsy (for disease confirmation) were used for analysis. Written informed consent was obtained from all the subjects. Inclusion criteria were that the pathology specimen must have been obtained by resection (preferably) or by multiple biopsies (>2) via stereotactic guidance. Fewer than two biopsies were not allowed because of the potential for sampling error. Histology was re-reviewed by a neuropathologist, blinded to the original diagnosis and type of radiation, in order to quantify the percentage of radiation necrosis and recurrent tumor. In order to avoid any training errors due to “mixed” pathologies on the same lesion the presence of RN was strictly defined as >80% RN and of recurrent tumor as >80% recurrent tumor (other “mixed” cases with varying proportions of RN and tumor recurrence were excluded). We identified a total of 42 cases, from 2006 to 2014 that followed this strict inclusion criterion. Our retrospectively analyzed brain tumor dataset comprised 22 primary (10 RN, 12 rBT) and 20 metastatic (8 RN, 12 rBT) cases. Patient MRIs were acquired at 3 Tesla. Images have an in-plane resolution of 0.8–0.93 mm/pixel. Slice thickness = 3–5 mm, TR = 400–750 ms, TE = 14–17 ms. The clinicopathologic characteristics of the brain tumor dataset have been summarized in Table 2.

#### Breast cancer dataset

Breast MRI data was prospectively collected in an Institutional Review Board-approved (IRB #02-13-42C), HIPAA-compliant study at the University of Pennsylvania (UPenn) between 2002 and 2007. Written informed consent was obtained from all subjects. Women without contraindication to MRI or gadolinium who presented with either a suspicious breast lesion or known malignancy prior to surgery were recruited to a larger single-institution study of MRI in the staging, diagnosis, and screening of breast cancer. Women who underwent neoadjuvant chemotherapy prior to surgery were excluded, as were women who had excisional biopsy prior to entry. From this data set we sub-selected women whose pathology revealed invasive cancer. Subjects whose images of the index lesion demonstrated substantial metallic artifact from prior biopsy were also excluded. This study examined MRI characteristics in 76 solid lesions from 65 patients for whom pathology results and, where applicable, ER, PR, and HER2 results were available. Reference standard diagnosis was made by histopathologic examination of tissue obtained by either core biopsy sampling or lumpectomy. Of the 76 lesions, 12 were benign fibroadenomas and 64 were invasive carcinomas. All of the carcinomas were immunohistochemically stained for hormone receptors and HER-2/neu. In cases in which staining for HER-2/neu was inconclusive, amplification was confirmed with fluorescence *in situ* hybridization. Of the 64 carcinomas, 21 were triple negative (ER−/PR−/HER2−) cancer, 18 were HER2+ (14 ER−/HER2+, 4 ER+/HER2+) cancer, and 25 were ER+ (ER+/HER2−) cancer. Patient MRIs were acquired at either 1.5 or 3 Tesla (Siemens Sonata or Trio, respectively, Malvern, PA). Imaging parameters for DCE-MRI varied over time and magnet type (in-plane resolution 0.20–0.70 mm/pixel, slice thickness 2–5 mm, TR = 7–26 ms, TE = 1.8–6.5 ms, flip angle 25–30 degrees. The clinicopathologic characteristics of the breast cancer dataset have been summarized in Table 3.

#### Lung cancer dataset

Two separate datasets of non-contrast Lung CT scans were prospectively collected in an Institutional Review Board-approved (IRB #02-13-42C), HIPAA-compliant study from two collaborating institutions: University Hospitals, Cleveland and Cleveland Clinic Foundation (CCF) in 2013 and 2014. Written informed consent was obtained for all the studies within the two cohorts. Histology was confirmed by anatomical pathologists at the respective institutions from the surgical specimen available for studies employed in both the cohorts. All patients underwent non-contrast CT scans prior to resection as part of routine care. Patients with multiple solitary nodules were excluded. Dataset 1 from University Hospitals, used as the training set, comprised 64 studies (31 adenocarcinomas and 33 granulomas). Dataset 2, from Cleveland Clinic, consisted of 56 cases (34 adenocarcinomas and 22 granulomas), and was used as an independent test set. The CTs were acquired using Siemens scanners with 2 mm slice thickness and 1 mm reconstruction. The tube voltage/current was 120 kV/150 mAs. The clinicopathologic characteristics of the lung cancer dataset have been summarized in Table 4.

#### Pre-processing

For the brain tumor cohort, the lesion ROIs were manually traced by an expert neuroradiologist, with over 25 years of experience, using the annotation tool in 3D Slicer after skull-stripping and intensity standardization. Intensity standardization^17^,^18^ is an essential pre-requisite when comparing image intensities across different acquisitions, as it allows the gray scale MR intensities to have a fixed tissue-specific meaning within the same imaging protocol and the same body region, and within the same patient. For the brain MRI cohort, the segmented regions included enhancing and non-enhancing neoplastic tissue with the exclusion of edema. The neuroradiologist annotated all the 2D slices that had visible lesions.

For the breast cancer and lung cancer cohort, the best representative section, which was a central section of either the DCE-MRI volume or the non-contrast CT scan, was annotated by a breast radiologist and a thoracic radiologist respectively, who were both blinded to the pathologic diagnosis. Both for lung and breast cohorts, the lesion boundary was manually delineated on the basis of the image that demonstrated the greatest lesion conspicuity from neighboring tissues, which was then used for subsequent analysis.

### Comparison of CoLlAGe with other popular texture features

While we qualitatively evaluated 3D CoLlAGe on a limited cohort of studies, quantitative analysis for all the three use cases in brain, breast, and lung, was restricted to 2D CoLlAGe, due to the variable slice thickness and anistropic MRI volumes of our retrospective studies. We compared the performance of 2D CoLlAGe with other state-of-the-art texture descriptors (i.e. Haralick, Gabor, HOG). Apart from CoLlAGe, we extracted a total of 584 2D texture features for the ROIs on a per-pixel basis, including 52 Haralick features, 432 Gabor features and 100 HOG descriptors. The Gabor filter bank consisted of six different frequency-shift values 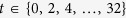, eight orientation parameter values 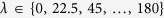, and 9 different variance settings, generating a total of 432 different filters. Each filter yielded a real and imaginary response, which were used to calculate the total response magnitude for every region of interest. HOG features were computed via pixel-wise gradient orientations obtained from the grayscale intensity differences in the region of interest. These orientations were then equally binned into *v* bins, 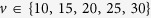, with each bin encompassing 36°, 24°, 18°, 14.4° or 12°. For Haralick and Gabor descriptors, the feature representations were obtained from every lesion by computing the median of feature values across all pixels within a lesion. Summary of parameters used for different feature sets is provided in Table 5.

### Comparison of CoLlAGe with expert diagnosis

To further demonstrate the efficacy of CoLlAGe in distinguishing similar appearing pathologies on imaging, we performed a human machine comparison for our brain (N = 42) and lung (N = 20) cohort. For both the cohorts, collaborating expert readers (board certified attending radiologists and pulmonologists) independently provided a diagnosis, which was then compared with the analysis from the CoLlAGe classifier. In both the human-machine comparison experiments, the expert readers were kept blinded to the pathology reports. The experts assigned a score between [0, 1] to each lesion, with 0 referring to a high confidence that the nodule is “benign” (radiation necrosis or granuloma), and 1 being “malignant” (recurrent tumor or adenocarcinoma). Similarly, probability scores were assigned by the Random Forest classifier using CoLlAGe features. Using the assigned probabilities we computed the areas under the respective receiver operating characteristic curves (AUCs).

### Experimental Evaluation

The feature sets were used to train a Random Forest (RF) classifier^19^, a boostrapped aggregation of multiple decision tree classifiers, in conjunction with **F**^2*D*^ to distinguish between the categories of interest. Wilcoxon’s rank sum test^20^ was employed to assess statistical significance and corrected for multiple comparisons for the experiments performed for the three use-cases. The sample size for the different experiments has been summarized in Table 6. In all our experiments, the RF classifier was used to assign every ROI into classes {+1, −1} based on the following classification tasks:

- **Experiment 1**: Distinguishing radiation necrosis from recurrent tumor on MRI,
- **Experiment 2**: Distinguishing triple negatives from other molecular subtypes of breast cancer (ER+, HER2+ and benign FA) on MRI,
- **Experiment 3**: Distinguishing adenocarcinoma from granuloma on non-contrast CT.

#### Experiment *E*_1_: Distinguishing Radiation Necrosis from Recurrent Tumor

We computed **F**^2*D*^ for every slice with expert-annotated ROI on the primary and metastatic brain tumor cohorts and employed the feature set in a RF classifier setting to distinguish RN from rBT such that slices from the same patient are either used for training or for testing. A total of 50 trees were used for training the RF classifier. 3-fold randomized cross-validation was used to train and evaluate classifier performance. This involved randomly splitting the entire dataset into 3 equally sized sets with 2 subsets used for classifier training and 1 subset used for independent evaluation. The diagnostic performance of each classifier trained with CoLlAGe and other comparative features was evaluated using average classification accuracy *β*^*Acc*^, computed over 150 iterations of cross-validation runs. Quantitative results across CoLlAGe and the other texture features were compared by computing *β*^*Acc*^ for every feature set at the operating point. Additionally, for a subset of studies we computed **F**^3*D*^ for qualitative visualization. Qualitative results for both 2D and 3D CoLlAGe feature representation were visualized as standard heatmaps where high CoLlAGe values were shown in red while blue represented low CoLlAGe values.

#### Experiment *E*_2_: Distinguishing Molecular Subtypes of Breast Cancer

**F**^2*D*^ was similarly computed for the slices with expert annotated ROI for the breast cancer cohort and was used to distinguish triple negative from the other breast cancer subtypes (ER+, HER2+) and benign fibroadenoma. A similar cross-validation technique was employed, along with a comprehensive comparison with other texture descriptors as given in *E*_1_.

#### Experiment *E*_3_: Distinguishing Adenocarcinomas from Granulomas

For every slice with the expert annotated ROI, **F**^2*D*^ was similarly computed for the lung cancer cohort and was used to distinguish adenocarcinomas from granulomas. Cross-validation, along with a comprehensive comparison with other texture descriptors as given in *E*_1_ was employed for the training set. This cross-validation strategy was employed for all the parameters as listed in Table 5. The model using the parameters that yielded the best results, for each category of feature descriptors, across the aggregated cross-validation runs was ‘locked down’, and then used to classify the cases in the independent test cohort.

### Parameter Sensitivity Analysis

**Figure 4:**
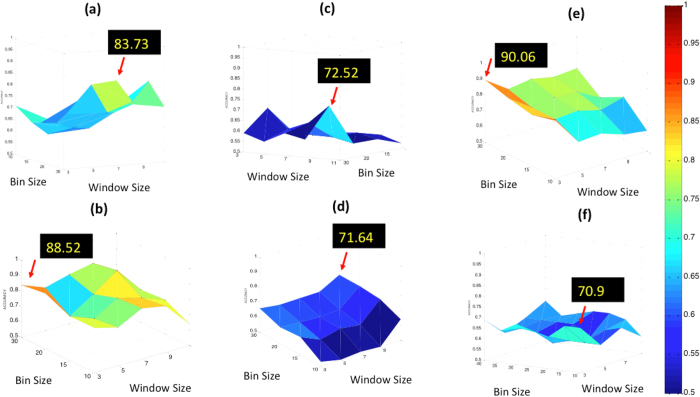
Parameter sensitivity analysis shown for CoLlAGe features for the six different classification experiments (**a**) RN vs rBT (primary brain tumors), (**b**) RN vs rBT (metastatic brain tumors), (**c**) TN vs ER+, (**d**) TN vs HER2+, (**e**) TN vs FA and (**f**) adenocarcinoma vs granuloma. The most optimal CoLlAGe parameters for each of the experiments were found to be as follows: (**a**) 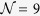, *v* = 30 (**b**) 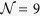, *v* = 20 (**c**) 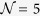, *v* = 30 (**d**) 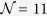, *v* = 30 (**e**) 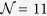, *v* = 30 (**f**) 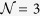, *v* = 10, respectively.

The sensitivity of CoLlAGe features was evaluated across its two key parameters, bin size of the entropy histogram (*v*) and neighborhood 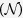 size for computing localized orientations. To account for smaller lesion sizes across the two cohorts, we restricted the 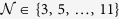. Bin sizes 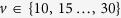 were additionally considered at regular bin intervals of 5 to evaluate variation in *β*^*Acc*^. We reported the variation in *β*^*Acc*^ as a function of 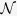 and *v. v* > 20, and 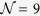 (Fig. 4) were found to be optimal parameters for the brain tumor cohort. Similarly, for the breast cancer cohorts (results not shown), the following pairs of parameters were found to be optimal: *v* = 30, and 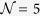 for TN versus ER+, *v* = 30, and 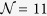 both for TN versus HER2+ and TN versus FA. For the lung cancer cohort, the best parameters were found to be *v* = 10, and 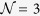. The bounds of the different parameters were selected in a way that accounted for boundary effects that would arise in case of relatively small lesions 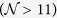, and when *v* > 30. Figure 4 shows the parameter sensitivity for all the different classification experiments using CoLlAGe.

## Results and Discussion

### Experiment *E*_1_: Distinguishing Radiation Necrosis from Recurrent tumor

**Figure 5:**
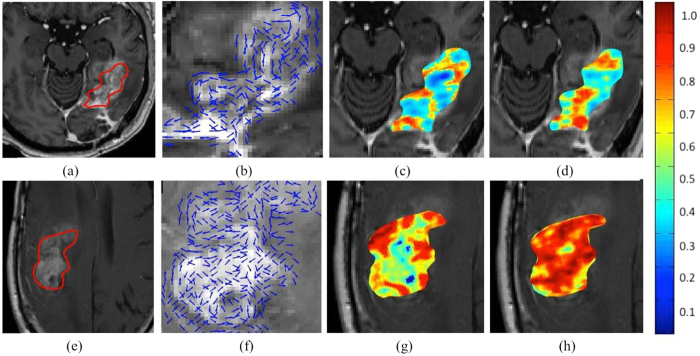
CoLlAGe entropy maps on brain tumor cases. Qualitative feature maps of 3D CoLlAGe visualized on Gd-T1 MRI for radiation necrosis (**a**) and recurrent tumor (**e**) respectively. The localized gradient orientations are shown in (**b**) and (**f**) for radiation necrosis and tumor recurrence, while the CoLlAGe heatmaps, 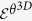 and 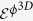 are shown in (**c**), (**d**) for radiation necrosis and in (**g**), (**h**) for tumor recurrence respectively. As evident, tumor recurrence has a higher density of high entropy regions both in (*X, Y*) as well as (*X, Y, Z*) directions than radiation necrosis.

Figure 5 shows the qualitative feature maps of 3D CoLlAGe on a Gd-T1 MRI for radiation necrosis (a) and recurrent tumor (e) respectively. The localized gradient field, *θ*^3*D*^, for radiation necrosis and recurrent tumor is shown in (b) and (e), while the CoLlAGe entropy heatmaps, 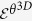 and 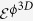 are shown in (c), (d) for radiation necrosis and in (g), (h) for tumor recurrence respectively. High CoLlAGe values are reflected in red, while blue reflects under expression of CoLlAGe values. As may be evidenced from the CoLlAGe heatmaps, tumor recurrence has an over-expression of CoLlAGe both in (*X, Y*) as well as (*X, Y, Z*) directions compared to radiation necrosis. The over-expression of CoLlAGe values may be reflective of the higher structural heterogeneity of recurrent tumor, owing to the presence of more varied tissue types and hypercellularity, as compared to radiation necrosis.

The quantitative results including comparison of the classification performance of 2D CoLlAGe with Haralick, Gabor, and HOG, using the best parameter settings, are shown in Table 7. The best classification accuracy obtained for the popular-texture features was reported to be between 50% to 65%, while CoLlAGe was found to perform significantly better (over 20% improvement in classification accuracy (*p*-value < 0.001)) with an accuracy of 83.79 ± 5.43% for primary cases, and 88.52% ± 3.93 for the metastatic brain tumor cohort. It has been previously shown that anomalies in brain tissue morphology are associated with directional patterns that can be captured by texture analysis. For example, gyrifications in gray matter create oriented spatial frequencies, that can be captured by wavelet-based features. Kovalev *et al*.^21^ have analyzed gradient and anisotropy properties of 3D texture in the context of neurodegenerative diseases. According to Georgiadia *et al*.^22^, brain lesion texture is correlated with presence and type of cancer cells. A recent study^23^ employed Haralick and wavelet texture features to distinguish radiation necrosis from metastatic brain tumor recurrence with a reported AUC of 94%. However, we believe that the results, reported on a per-slice basis, may have been affected by the classifier being contaminated by slices from the same patient being used both within the training as well as testing sets during classification. It is worth noting that the diagnostic accuracy of distinguishing radiation necrosis from tumor recurrence by an expert radiologist on visual inspection of MRI has been reported to be between 50–60%^24^.

### Experiment *E*_2_: Distinguishing Molecular Sub-types of Breast Cancer

**Figure 6:**
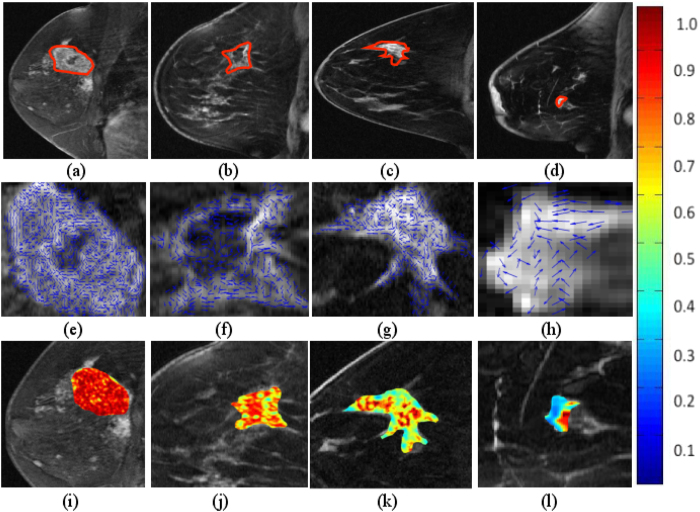
CoLlAGe entropy maps on breast cancer cases. 2D DCE-MRI (1.5 Tesla) scan for TN+ (**a**), HER2+ (**b**), ER+ (**c**), and FA (**d**) with the lesion outlined in red. 4(**e**), (**f**), (**g**), (**h**) represent localized gradient orientations, while 4(**i**), (**j**), (**k**), (**l**) represent CoLlAGe heatmaps for the corresponding lesion on (**a**), (**b**), (**c**) and (**d**), where red represents higher while blue represents low CoLlAGe values.

Figure 6 shows the qualitative 2D CoLlAGe feature maps for each of the breast cancer sub-types, TN (i), HER2+ (j), ER+ (k), and FA (l). Higher CoLlAGe values are reflected in red, while blue reflects low CoLlAGe values. The corresponding localized orientations (*θ*^2*D*^) for TN, HER2+, ER+ and FA are shown in Fig. 6(e), (f), (g) and (h) respectively. It is interesting to note that the gradient field was found to be more disordered across cancer sub-types, as compared to benign FA. Similar to Experiment *E*_1_, a marked difference in CoLlAGe values was observed across different sub-types of breast cancer (TN, HER2+, ER+, and FA), suggesting that CoLlAGe may potentially be capturing local anisotropic differences in micro-structures on imaging that are otherwise not visually appreciable.

Table 7 shows the average *β*^*Acc*^ values obtained over 150 iterations of a 3 fold-cross validation via a RF classifier. *β*^*Acc*^ values obtained from CoLlAGe in the breast cancer cohort significantly outperformed (*p*-value < 0.001) the other state-of-the-art texture descriptors (Haralick, Gabor, HOG), with an improvement of ≈10% for majority of the 3 classification tasks (TN versus ER+, TN versus HER2+, TN versus FA). Our results resonate with the findings reported in Agner *et al*.^25^ that used a similar cohort of breast DCE-MRI studies to distinguish different sub-types of breast cancer using a novel texture kinetic approach. Similar to Agner *et al*., the most prominent difference, both in qualitative and quantitative performance of CoLlAGe, is reported between FA, a benign condition, from TN, the most aggressive sub-type of breast cancer with *β*^*Acc*^ of 90.06 ± 4.38. It is worth noting that currently radiologists are unable to distinguish TNs from FAs on a routine MRI^26^,^27^,^28^.

### Experiment *E*_3_: Distinguishing Adenocarcinomas from Granulomas

**Figure 7:**
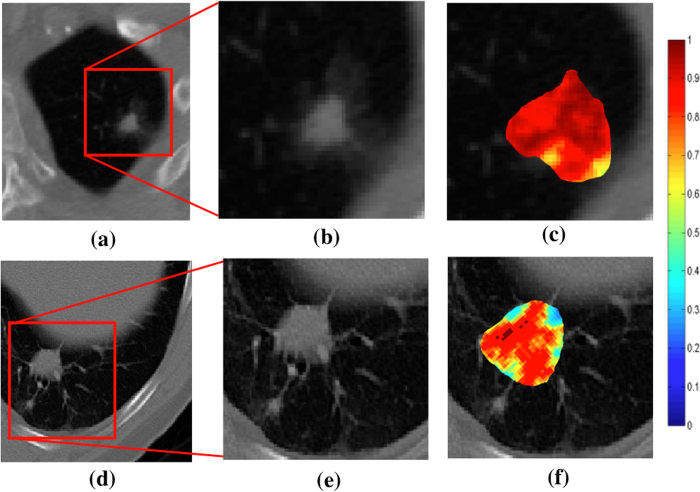
CoLlAGe entropy maps on lung cancer cases. Qualitative feature maps of 2D CoLlAGe visualized on non-contrast lung CT for (**a**) adenocarcinoma and (**d**) granuloma shown in (**c**) and (**f**) respectively. Adenocarcinomas have higher CoLlAGe entropy values as compared to granulomas. Red represents higher while blue represents low CoLlAGe values.

Figure 7 shows the qualitative 2D CoLlAGe feature maps for a representative non-contrast CT image with pathologically proven adenocarcinoma (a) and granuloma (d), respectively. Higher CoLlAGe values are reflected in red, while blue reflects low CoLlAGe values. It may be observed that representative adenocarcinoma lesion has higher density of larger CoLlAGe entropy values as compared to the granuloma sample. Table 7 shows the average *β*^*Acc*^ values obtained over 150 iterations of a 3 fold-cross validation via a RF classifier. Using the locked-down classifier, CoLlAGe features showed the best classification results for the test set (69.8%).

Tumor heterogeneity has been previously shown to be associated with non-small cell lung carcinoma^29^. This heterogeneity can be attributed to the hypoxic microenvironment^30^. The subtle differences in the hypoxia-related heterogeneity as suggested in ref. 29 is perhaps manifested in the differential expression of CoLlAGe entropy. Dennie *et al*.^31^ have reported an AUC of 0.90 in distinguishing the two conditions. The texture analysis in ref. 31, using Haralick features, has yielded higher accuracy than FDG-PET/CT in distinguishing the two pathologies. However, the approach in ref. 31 has not been validated on an independent test set. Besides, there is no clear qualitative evidence of the Haralick features visibly distinguishing the two pathologies.

### Human-Machine Comparison Results

On the hold-out lung cohort, the AUCs for the two experts were found to be 0.68, and 0.54 respectively. Using the CoLlAGe features, the associated AUC was computed as 0.78. For the brain tumor studies, AUCs for the two independent readers were 0.75 and 0.58 respectively, while the same for a CoLlAGe-based classifier was computed to be 0.80. To the best of our knowledge, no radiomics-based work recently has demonstrated such a rigorous and comprehensive human-machine reader comparison across multiple disease sites.

## Concluding Remarks

We presented a radiomic feature descriptor, Co-occurrence of Local Anisotropic Gradient Orientations (CoLlAGe), that captures higher order co-occurrence patterns of local gradient tensors at a voxel level to distinguish disease phenotypes that have similar morphologic appearances. We employed three clinically challenging datasets to evaluate the efficacy of CoLlAGe, distinguishing (a) radiation necrosis, a relatively benign effect of radiation, from tumor recurrence on T1-w MRI in brain tumors, (b) different molecular sub-types of breast cancer on DCE-MRI and (c) adenocarcinomas from granulomas on non-contrast CT. Additionally, we compared performance of CoLlAGe with other state-of-the-art texture descriptors (Haralick, Gabor, Histogram of Gradient orientations) as well as across two expert readers (for two use-cases), and demonstrated that CoLlAGe has significantly better classification accuracy than the other texture descriptors as well as the expert readers. Across the cross-validation and testing stages, CoLlAGe outperformed other texture features in 20 out of 21 comparative experiments (Table 7). Our results, on all three cohorts, seem to suggest that CoLlAGe has the potential to serve as a powerful radiomic descriptor in distinguishing similar appearing pathologies on imaging.

Nevertheless, there are a few limitations to our study. Firstly, CoLlAGe was only compared against three popularly used texture features (Haralick, Gabor, and HOG). Secondly, based on our parameter sensitivity analysis, it appears that parameter selection may be an important consideration when employing CoLlAGe for a specific problem. In this paper, histogram representation was used to collate CoLlAGe values to classify every image within a random forest classifier. However, the choice of feature representation and classification methods is flexible and can be modified depending on the specific application. Future work will focus on (1) rigorously evaluating efficacy of CoLlAGe across other texture features on a larger cohort of multi-institutional studies, and (2) identifying a domain-independent parameter selection strategy to evaluate robustness of CoLlAGe.

## Additional Information

**How to cite this article**: Prasanna, P. *et al*. Co-occurrence of Local Anisotropic Gradient Orientations (CoLlAGe): A new radiomics descriptor. *Sci. Rep.* **6**, 37241; doi: 10.1038/srep37241 (2016).

**Publisher’s note:** Springer Nature remains neutral with regard to jurisdictional claims in published maps and institutional affiliations.

## Figures and Tables

**Figure 1:**
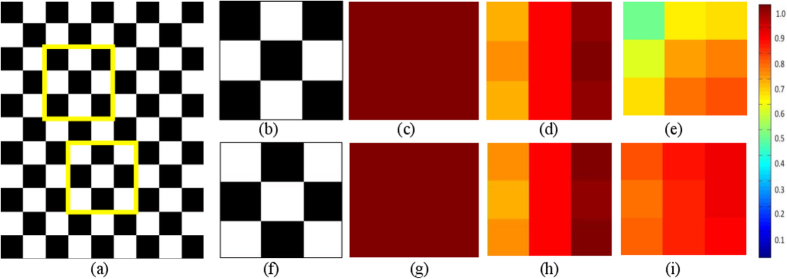
Comparison of different feature representations for two similar-appearing toy images. (**a**) A synthetic checkerboard image. Two 3 × 3 patches (*P*_1_, *P*_2_) obtained from the checkerboard image are shown in (**b**) and (**f**) respectively such that *P*_2_ = ~(*P*_1_). The corresponding feature representations for *P*_1_ and *P*_2_ are shown in (**c**) and (**g**) for Haralick entropy, (**d**) and (**h**) for Gabor (*t* = 4, *λ* = 22.5), and (**e**) and (**i**) for CoLlAGe respectively.

**Table 1:** List of commonly used notations, symbols and acronyms in this paper.

Symbol	Description	Symbol	Description
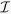	MRI scene	*β*^*Acc*^	Average accuracy
*C*	Grid of MRI voxels	*c*	Spatial location of pixel/voxel of interest
*f(c*)	MRI signal intensity at *c*	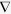	Spatial derivative
∂ *f*_*X*_ (*c*)	Gradient magnitude along *X*-axis	∂ *f*_*Y*_ (*c*)	Gradient magnitude along *Y*-axis
∂ *f*_*Z*_ (*c*)	Gradient magnitude along *Z*-axis	*W*	Local neighborhood around central location
*k*	Index of voxels/pixels of interest, *k* ∈ 1, …, |*C*|	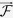	Vector gradient matrix
*v*	Bin size	*ω*	Discretization factor
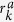	Principal component in 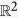 , *a* ∈ *X, Y*	*ψ*_*n*_(*c*_*k*_)	Principal component in 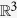 , *n* ∈ *X, Y, Z*
*θ*^2*D*^	Dominant orientation computed from 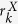 , 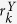	*θ*^3*D*^	Dominant orientation computed from *ψ*_*X*_, *ψ*_*Y*_
*ϕ*^3*D*^	Dominant orientation computed from *ψ*_*X*_, *ψ*_*Y*_, *ψ*_*Z*_	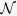	Window size dimensions (pixels/voxels)
*N*	Window size for co-occurrence computation	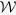	Co-occurrence matrix neighborhood
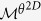	Co-occurrence matrix computed from *θ*^2*D*^	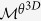	Co-occurrence matrix computed from *θ*^3*D*^
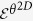	Entropy map computed from 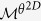	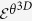	Entropy map computed from 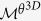
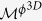	Co-occurrence matrix computed from *ϕ*^3*D*^	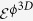	Entropy map computed from 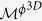
**F**^2*D*^	2D CoLlAGe feature vector	**F**^3*D*^	3D CoLlAGe feature vector
UH	University Hospitals, Cleveland	CCF	Cleveland Clinic Foundation
UPenn	University of Pennsylvania	RN	Radiation Necrosis
rBT	Recurrent Brain Tumor	TN	Triple Negative
ER	Estrogen Receptor	HER2	Human Epidermal growth factor Receptor 2
FA	Fibroadenoma	RF	Random Forest
PET	Positron Emission Tomography	CT	Computed Tomography
FDG	Fluorodeoxyglucose	Gd	Gadolinium

**Table 2:** Clinicopathologic characteristics of brain tumor studies.

	Primary Brain tumors	Metastatic Brain Tumors
Number of patients (samples)	22 (10 RN, 12 rBT)	20 (8 RN, 12 rBT)
Gender	12 F, 10 M	5 F, 15 M
Age (y, range)	53 (33–75)	50 (37–65)

**Table 3:** Clinicopathologic characteristics of breast cancer studies.

	Fibroadenoma	HER2+	ER+	Triple Negative
Number of patients (samples)	9	14	23	19
Number of lesions	12	18	25	21
Age (y, range)	46 (32–60)	50 (38–63)	45 (32–70)	51 (32–68)
Pre-menopausal (n)	5	5	15	9
Post-menopausal (n)	2	9	1	7
Peri-menopausal (n)	2	0	7	3
Lymph Node Positive Studies	0	1	8	3

**Table 4:** Clinicopathologic characteristics of lung studies.

	Adenocarcinomas	Granulomas
Number of patients (samples)	65	55
Gender	39 F, 26 M	25 F, 30 M
Age (y, range)	67 (40–85)	58 (18–84)

**Table 5:** Summary of features and feature parameters used in this work.

Descriptor	#	Feature setting
Haralick	52	Window size 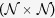 ; 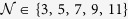 ; *θ* ∈ [0 180°]
HOG	100	*v* ∈ {10, 15, 20, 25, 30}; *κ* = 360°/*v*
Gabor	432	*t* ∈ {0, 2, 4, …, 32}; *λ* ∈ {0, 22.5, …, 180}; 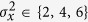 , 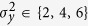
CoLlAGe	500	Window size 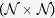 ; 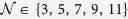 ; *v* ∈ {10, 15, 20, 25, 30}

A total of 26 parameters (across different features) were compared and evaluated in terms of their classification performance, for each of the three experiments in brain, breast and lung cancers.

**Table 6:** List of studies employed in this work for three different clinical problems in brain, breast, and lung cancers.

Cancer Site	Cohort Size (Site)	Clinical challenge	Classification task	Number of samples
Brain Tumors	42 (UH)	RN versus rBT	Primary tumor cohort	10 RN, 12 rBT
			Metastatic tumor cohort	8 RN, 12 rBT
Breast Cancer	65 studies (UPenn)	Distinguish subtypes of breast cancer	TN-ER	21 TN, 25 ER+
			TN-HER2+	21 TN, 18 HER2+
			TN-FA	21 TN, 12 FA
Lung Cancer	120 studies (UH, CCF)	Distinguish granuloma from adenocarcinoma	Training Set (UH)	31 adenocarcinoma, 33 granuloma
			Test Set (CCF)	34 adenocarcinoma, 22 granuloma

The data acquisition sites include University Hospitals, Cleveland (UH), University of Pennsylvania (UPenn) and Cleveland Clinic (CCF).

**Table 7:** *β* ^*Acc*^ for 2D CoLlAGe and the comparative strategies (Haralick, Gabor, and HOG) obtained across 150 iterations of 3-fold cross validation in a random forest classifier setting for the brain and breast cancer use-cases, as well as for independent training and test dataset for the lung cancer use-case.

Classification Task	Haralick	HOG	Gabor	**CoLlAGe**
RN vs rBT (GBM)	62.19 ± 0.99	60.62 ± 3.21	59.68 ± 5.8	**83**.**73** ± **5**.**43**
RN vs rBT (Metastatic)	63.83 ± 2.42	72.99 ± 1.35	59.45 ± 1.73	**88**.**52** ± **3**.**93**
TN vs ER+	60.5 ± 4.49	68.49 ± 3.8	62.37 ± 4.29	**72**.**52** ± **5**.**19**
TN vs HER2+	54.69 ± 5.19	60.18 ± 4.08	62.67 ± 3.97	**71**.**64** ± **6**.**13**
TN vs FA	78.37 ± 3.75	72.7 ± 4.41	68.1 ± 1.85	**90**.**06** ± **4**.**38**
Adenocarcinoma vs Granuloma (Training)	75.5 ± 5	67.7 ± 5.4	66.2 ± 4.4	**70**.**9** ± **7**.**1**
Adenocarcinoma vs Granuloma (Testing)	69.6	67.3	62.4	**69**.**8**
